# Controlling Supramolecular Assembly through Peptide Chirality

**DOI:** 10.1021/acsami.5c14913

**Published:** 2025-11-25

**Authors:** Manosree Chatterjee, Itzhak Grinberg, Santu Bera, Dana Cohen-Gerassi, Oren Ben-Zvi, Iftach Yacoby, Moran Aviv, Lihi Adler-Abramovich

**Affiliations:** † Department of Oral Biology, The Goldschleger School of Dental Medicine, The Gray Faculty of Medical and Health Sciences, 26745 Tel Aviv University, Tel Aviv 6997801, Israel; ‡ The Jan Koum Center for Nanoscience and Nanotechnology, Tel Aviv University, Tel Aviv 6997801, Israel; § The Center for the Physics and Chemistry of Living Systems, Tel Aviv University, Tel Aviv 6997801, Israel; ∥ New Environmental School (NES), Tel Aviv University, Tel Aviv 6997801, Israel; ⊥ Department of Materials Science and Engineering, Tel Aviv University, Tel Aviv 6997801, Israel; # School of Plant Sciences and Food Security, The George S. Wise Faculty of Life Sciences, Tel Aviv University, Tel Aviv 6997801, Israel; ∇ School of Mechanical Engineering, Afeka Tel Aviv Academic College of Engineering, Tel Aviv 6910717, Israel

**Keywords:** hydrogel, peptide, self-assembly, phase transition, nanostructures, enantiomer

## Abstract

Peptide-based self-assembled hydrogels are promising materials for diverse applications due to their biocompatibility, tunable mechanical properties, and ability to form nanostructured networks via noncovalent interactions. One of the most extensively studied hydrogelators, fluorenylmethyloxycarbonyl-diphenylalanine (Fmoc-FF), rapidly self-assembles into a 3D hydrogel capable of encapsulating enzymes and proteins, making it an attractive candidate for drug delivery applications and the protection of oxygen-sensitive biomolecules. However, its fast gelation results in heterogeneous structures and low-density cavities, limiting its uniformity and complicating the handling. Chirality plays a critical role in peptide self-assembly, yet its impact on hydrogel functionality remains underexplored. Here, we investigate how chirality influences the self-assembly kinetics, morphology, and structural properties of all four enantiomeric forms of Fmoc-FF. Using a range of analytical techniques, we tracked the morphological transitions from monomers to supramolecular nanostructures. Hydrogels formed from homoenantiomers displayed greater rigidity and faster gelation, while heteroenantiomeric systems exhibited a slower, three-phase transition from turbid nanospheres to transparent fibrillary gels. This slower gelation may be advantageous for controlled encapsulation, allowing for homogeneous distribution of the cargo. Finally, all enantiomeric hydrogels effectively prevented oxygen diffusion through their nanofiber networks, allowing H_2_ production by the oxygen-sensitive enzyme hydrogenase, which was encapsulated within the hydrogels. These findings highlight the potential of enantiomeric design in developing peptide hydrogels for various applications, particularly the encapsulation of small molecules and large proteins as well as oxygen-sensitive processes.

## Graphical abstract

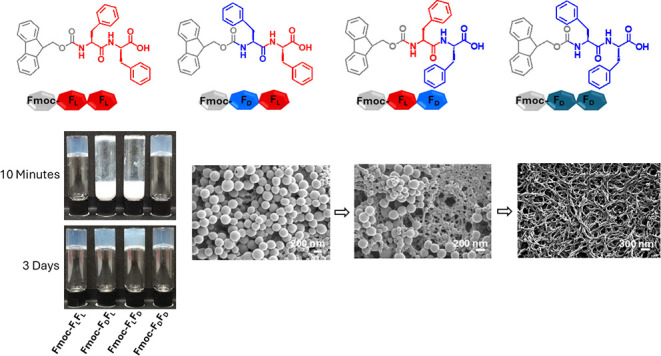

## 1 Introduction

Over the past two decades, peptide self-assembly has emerged as a powerful strategy for constructing supramolecular functional materials.^1,2^ This approach draws inspiration from natural self-assembly processes, such as DNA double-helix formation, protein folding, and phospholipid-driven membrane synthesis. Self-assembly is a spontaneous organization of molecules into ordered structures stabilized by weak, noncovalent interactions, which maintain the system in a low-energy state.^3,4^

Among biomolecular building blocks, short peptides, particularly dipeptides, have received considerable attention due to their structural simplicity, ease of modification, and cost-effective synthesis.^5^ Specifically, peptide-based hydrogels, formed through the hierarchical self-assembly of such peptides into nanofibrous networks, represent a versatile class of soft materials. Their biocompatibility, tunable architecture, and responsiveness to environmental stimuli make them attractive for various biomedical applications, including controlled drug release and 3D cell culture scaffolds.^6^

An extensively studied example is diphenylalanine (FF), the core recognition motif of the Alzheimer’s β-amyloid peptide, which readily forms ordered structures via aromatic stacking.^7–9^ The self-assembly of FF can be enhanced by introducing aromatic N-terminal protecting groups such as fluorenylmethyloxycarbonyl (Fmoc), one of the most commonly used groups in peptide synthesis.^10^ The Fmoc moiety promotes assembly through a combination of hydrogen bonding involving its carbonyl group, as well as π–π stacking and hydrophobic interactions mediated by the aromatic fluorenyl ring.^11^ Fluorenylmethyloxycarbonyl-diphenylalanine (Fmoc-FF) peptide hydrogels uniquely exhibit the ability to encapsulate and protect oxygen-sensitive enzymes by specifically encaging O_2_ molecules within their nanofibrillar network, thereby significantly limiting oxygen diffusion and preserving enzymatic activity under ambient conditions.^12^ Despite extensive efforts to understand the underlying mechanisms of peptide assembly, achieving precise control over the process remains a central challenge in tailoring the structural and functional properties of the resulting materials.^13^

Chirality, a fundamental geometric property of molecules, is widely present in biological building blocks and plays a crucial role in the formation of self-assembled polymers. In nature, this is exemplified by the exclusive use of l-amino acids in proteins and d-sugars in polysaccharides.^14^ However, the molecular mechanisms driving the preferential selection of a single chirality and the specific interactions among chiral molecules in natural biopolymers remain poorly understood.^15^

Chirality profoundly influences the structure, properties, and functions of biomolecular assemblies. For instance, l-amino acids typically taste bitter, while their d-counterparts often taste sweet.^16^ In the context of self-assembly, chirality can determine both the ability of peptides to assemble and the morphology of the resulting structures. Marchesan et al. demonstrated that the tripeptides valine-phenylalanine-phenylalanine and phenylalanine-phenylalanine-valine fail to self-assemble in their all-l form, but substitution of the N-terminal residue with the corresponding d-enantiomer yields self-supporting hydrogels with distinct morphologies.^17^ Similarly, Arakawa et al. and Zhang et al. showed that altering a single chiral center in Fmoc-α-methyl-diphenylalanine^18^ and ferrocene-diphenylalanine^19^ peptides significantly impacts self-assembly and hydrogel morphology. Marchesan and co-workers also investigated eight enantiomeric combinations of the proline-phenylalanine-phenylalanine tripeptide, revealing diverse nanostructures and fibrillization behaviors, highlighting the structural and functional diversity imparted by stereoisomerism.^20^ Importantly, Adams and co-workers further showed that all enantiomers, diastereomers, and even racemates of functionalized dipeptides can form gels with distinct supramolecular morphologies and gelation kinetics, underscoring chirality as a key design parameter in supramolecular materials.^21^

While significant efforts have focused on modifying peptide structure to tailor supramolecular architectures,^22–24^ less attention has been paid to how chirality influences the kinetics of self-assembly, a key parameter that governs material properties and biological functionality.^25^ Zelenovskiy et al. examined how enantiomeric FF peptides affect the kinetics of microtube growth.^26^ Kralj et al. further demonstrated that combining l- and d-FF enantiomers allows control over supramolecular organization, including through halogenation of heteroenantiomeric systems.^27^ Beyond equilibrium systems, recent work by Saile et al. revealed that chirality can also dictate chemically driven, nonequilibrium self-assembly pathways, where the handedness of the activating agents modulates the reaction lifetime, assembly dynamics, and downstream cascade reactivity.^28^ Given the strong influence of chirality on the structural and functional properties of peptide assemblies, we hypothesized that it would also play a critical role in controlling the self-assembly kinetics of short peptides.^29^

Here, we investigated all four enantiomeric combinations of the minimalistic Fmoc-FF dipeptide hydrogelator to develop tunable self-assembly systems. The self-organization pathway, from the initial metastable nucleation phase to the formation of a stable, ordered fibrillary network, was systematically explored. We further conducted comparative analysis of the physical, mechanical, and chemical properties of the resulting enantiomeric hydrogels. The remarkable degree of kinetic control achieved highlights the critical role of chirality in directing the self-assembly behavior of short peptide-based materials. These hydrogels hold promise for diverse biomedical applications, including efficient encapsulation platforms for sensitive bioactive molecules and self-supporting matrices for tissue engineering.

## 2 Results and Discussion

### 2.1 Macroscopic and Microscopic Characterization of the Four Fmoc-FF Enantiomeric Hydrogels

Four Fmoc-FF enantiomeric variants were designed, namely, Fmoc-F_L_F_L_, Fmoc-F_D_F_L_, Fmoc-F_L_F_D_, and Fmoc-F_D_F_D_ (Figure 1a–d). The self-assembly of the Fmoc-FF enantiomeric variants was initiated by the solvent switch method.^30,31^ Decreasing the solubility by adding water to each peptide stock solution in DMSO triggered the self-assembly. Upon dilution in water, the clear stock solutions instantly produced a turbid white suspension, which gradually gelled and became transparent. We closely monitored the gelation process during self-assembly using a turbidity assay (Figure 1e).^32^ While the gelation duration of the Fmoc-F_L_F_L_ and Fmoc-F_D_F_D_ dipeptides was less than 10 min, the gelation of the heteroenantiomers Fmoc-F_D_F_L_ and Fmoc-F_L_F_D_ was longer and proceeded for more than one and 4 h, respectively. During the gelation process, three noticeable colorimetric stages were detected: milky-white, partially opaque, and transparent. These stages were observed only for the heteroenantiomers, suggesting slower kinetics of self-assembly. The absorbance of the hydrogels at 405 nm also correlated to these observations (Figure 1f). The turbid liquid state of the homoenantiomeric hydrogels persisted for a few seconds, in contrast to more than four h in the case of Fmoc-F_L_F_D_. The faster self-assembly of the homoenantiomeric peptides might be attributed to the higher molecular affinity of the building blocks compared to the heteroenantiomers.

**Figure 1:**
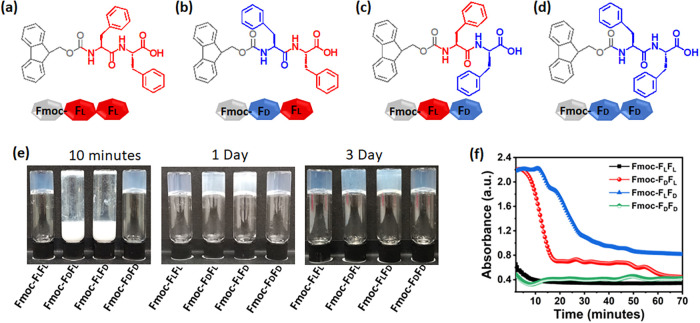
Peptide enantiomer assembly into hydrogels. (a–d) Molecular structures of the tested peptides. (a) Fmoc-F_L_F_L_, (b) Fmoc-F_D_F_L_, (c) Fmoc-F_L_F_D_, and (d) Fmoc-F_D_F_D_. (e) Macroscopic view of gelation kinetics using an inverted vial assay. (f) Absorbance of the peptides at 405 nm over time.

The assembly into different structural morphologies was studied by systematically varying the DMSO-to-water ratio and peptide concentration in 96-well plates. The peptide concentration was varied in the range of 0.2–10 mg/mL in different DMSO/water ratios from 10% to pure DMSO (Figure 2).^33,34^ The plates were sealed to avoid evaporation and allowed to equilibrate for 24 h at room temperature before imaging.^33^ The distinct self-assembly properties and critical gelation concentrations (CGC) were determined using optical microscopy.^35^ In the entire range of peptide and solvent concentrations, the homoenantiomeric peptides showed either a liquid phase or a very dense fibrillar gel phase, whereas the heteroenantiomers were present in several phases, namely, liquid, turbid gel, flower-like organization of fibrillary gel, needle-like organization of fibrillary gel, and crystalline phases (Figure 2). The CGC value was found to be 1 mg/mL in the DMSO range of 10–50% for the homoenantiomers, while the heteroenantiomeric hydrogels showed a lower CGC value of 0.2 mg/mL in 20–40% DMSO, even though the gelation time was longer. At any concentration within the range of gel phase, above the CGC value to 10 mg/mL in 60% DMSO, the homoenantiomeric peptides displayed a dense compact fibrillary phase and macroscopically transparent hydrogels (Figure 2a,d). However, in the heteroenantiomeric hydrogels, the dense, turbid fibrillar morphology appeared at lower DMSO ratios (10–30%) and specifically at higher peptide concentrations (Figure 2b,c). With a gradual increase of the DMSO ratio, turbidity disappeared, and a very organized fibrillary hydrogel with a flower-like structure was formed (Figure 2bii,biv,cii,civ). These structures became thicker and larger with an increase in peptide concentration. At a critical point of the peptide concentration and DMSO ratio, a transformation of the fibrillary structure into crystalline morphology was detected. A distinct bundle of needle-like fiber morphology and crystalline transformation was noticed at 6–10 mg/mL Fmoc-F_D_F_L_ in 60% DMSO after 48 h and in 70% DMSO after 72 h, respectively (Figure 2bv,bvi). At a critical ratio (8–10 mg/mL 60% DMSO), a transition of the crystalline structure was observed for Fmoc-F_L_F_D_ after 14 days (Figure 2cv,cvi). Regardless of the identity of the enantiomers, at a DMSO ratio >70%, all peptides remained in the solution state, and no gelation was observed.^33^ This phenomenon indicates that the peptides are highly soluble at a high DMSO concentration, which hinders the nucleation and eventually the self-assembly process.

**Figure 2:**
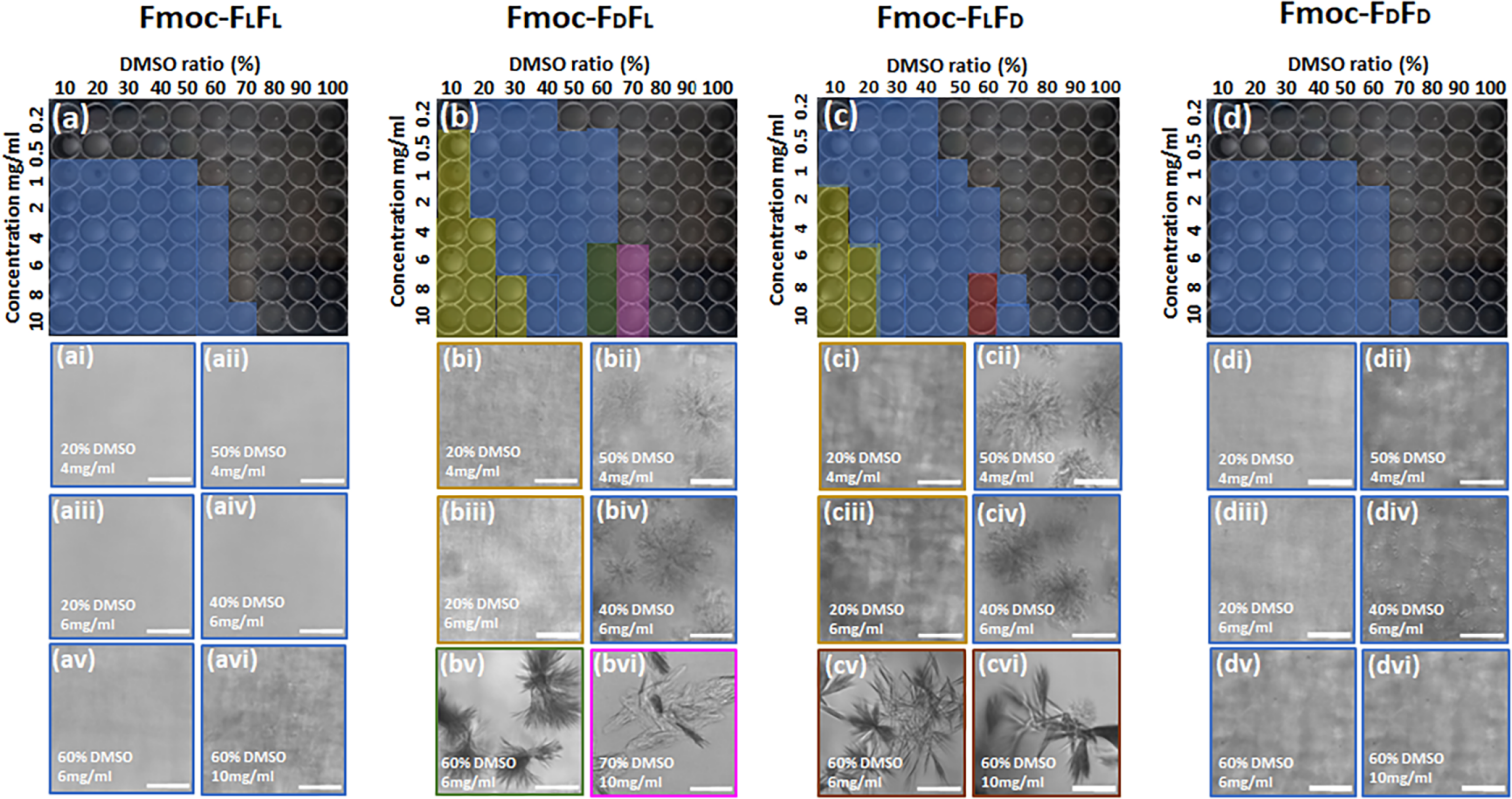
Structural study of the four Fmoc-FF enantiomeric hydrogels. (a–d) Transition from the fibrillar gel to crystal structure for different peptide concentrations in serial DMSO/water ratios in a 96-well plate. Yellow: opaque gel, blue: fibrillary gel, red, green, and pink: crystals of different morphologies. (ai–dvi) Optical microscopy images of the four Fmoc-FF enantiomeric hydrogels at various peptide concentrations and DMSO percentages. (ai–avi) Fibrillary structure of Fmoc-F_L_F_L_ in the following peptide and DMSO ratios: (ai) 4 mg/mL in 20%, (aii) 4 mg/mL in 50%, (aiii) 6 mg/mL in 20%, (aiv) 6 mg/mL in 40%, (av) 6 mg/mL in 60%, and (avi) 10 mg/mL in 60%. (bi-bvi) Phase transition of Fmoc-F_D_F_L_ from opaque gel at low DMSO ratio: (bi) 4 mg/mL in 20% and (biii) 6 mg/mL in 20%, to distinct fibrillary structures at higher DMSO ratio: (bii) 4 mg/mL in 50% and (biv) 6 mg/mL in 40%, to crystal formation at high DMSO and peptide ratios: (bv) 6 mg/mL in 60% and (bvi) 10 mg/mL in 70%. (ci–cvi) Phase transition of Fmoc-F_L_F_D_ from opaque gel at low DMSO ratio: (ci) 4 mg/mL in 20% and (ciii) 6 mg/mL in 20%, to distinct fibrillary structures at higher DMSO ratio: (cii) 4 mg/mL in 50% and (civ) 6 mg/mL in 40%, to crystal formation at high DMSO and peptide ratios: (cv) 6 mg/mL in 60% and (cvi) 10 mg/mL in 60%. (di-dvi) Fibrillary structure of Fmoc-F_D_F_D_ at the following peptide and DMSO ratios: (di) 4 mg/mL in 20%, (dii) 4 mg/mL in 50%, (diii) 6 mg/mL in 20%, (div) 6 mg/mL in 40%, (dv) 6 mg/mL in 60%, and (dvi) 10 mg/mL in 60%. Scale bar = 100 μm.

### 2.2 Real-Time Monitoring of Self-Assembly Processes and Phase Transition

The peptides, specifically the heteroenantiomeric peptides, demonstrated numerous morphologies under different conditions. To unveil the mechanism of phase transition during self-assembly, we utilized time-lapse optical microscopy imaging. For the homoenantiomeric hydrogels, the sphere phase was initially observed in the turbid solution, but within a minute, it became a stable gel (Figure S1 and Movies S1 and S2). Apart from the sphere phase, we were unable to detect other structural morphologies or transition states in the homoenantiomeric hydrogels, as the assembly into the fibril final state was very rapid. Using optical microscopy, the heteroenantiomeric peptides at 10 mg/mL in 60% DMSO were observed to form crystalline structures, which were chosen for further evaluation. The turbid phase of the peptide hydrogel was sealed inside a glass capillary to prevent evaporation and studied by using optical microscopy (Figure 3). In the case of both heteroenantiomeric peptides, spheres were observed throughout the capillary at the beginning of the self-assembly process. Similar spherical structures were previously reported for the self-assembly of Fmoc-l-phenylalanine-α-methyl-l-phenylalanine and Fmoc-d-phenylalanine-α-methyl-l-phenylalanine.^18^ Gradually, a fibrillary phase started to nucleate at different points in the capillary after 1 h of Fmoc-F_D_F_L_ and 10 min of Fmoc-F_L_F_D_ self-assembly (dotted red circles in Figure 3a,f, respectively). The transition kinetics were different for the two heteroenantiomer peptides. In both peptides, as the network of fibrils grew, the disappearance of the spheres was observed in their vicinity, leading to the transformation into filamentous structures over time. The sphere phase was significantly longer in the case of Fmoc-F_D_F_L_, which completely transformed into fibers within a very short time (Movie S3). In contrast, the spheres of Fmoc-F_L_F_D_ slowly transformed into fibers, and both the sphere and fiber phases coexisted for over 100 min (Movie S4). A possible explanation for this difference may be nucleation occurring at different focal planes, so only filaments within the chosen plane were viewed. In Fmoc-F_L_F_D_, this creates the impression that fiber elongation stops as spheres vanish, whereas rheology indicates continued nanofibril growth below the optical resolution (Figure 6b,c). The additional aggregates observed in Fmoc-F_D_F_L_ likely reflect sporadic bundling of nanofibrils into larger filaments captured in the field of view. Finally, the filamentous aggregates replaced all of the spheres. Moreover, we observed that the spreading of filaments depended on the availability of the surrounding spheres, which assembled on the growing filament template. These findings raise the hypothesis that the spheres were interacting with each other on the growing fibers. Different sizes of filamentous or fibrillar aggregates with identical morphology were observed in the entire capillary for individual heteroenantiomeric peptides, possibly due to the localized availability of the spheres, supporting the hypothesis. Importantly, high-resolution scanning electron microscopy (HRSEM) images of samples prepared from both the transparent and filamentous regions revealed comparable nanofiber morphology, indicating that the filamentous aggregates represent bundles of nanofibers rather than a distinct heterogeneous phase. The HRSEM images presented in Figure 3e,j were obtained from hydrogels formed in glass vials and are representative of the overall morphology.

**Figure 3:**
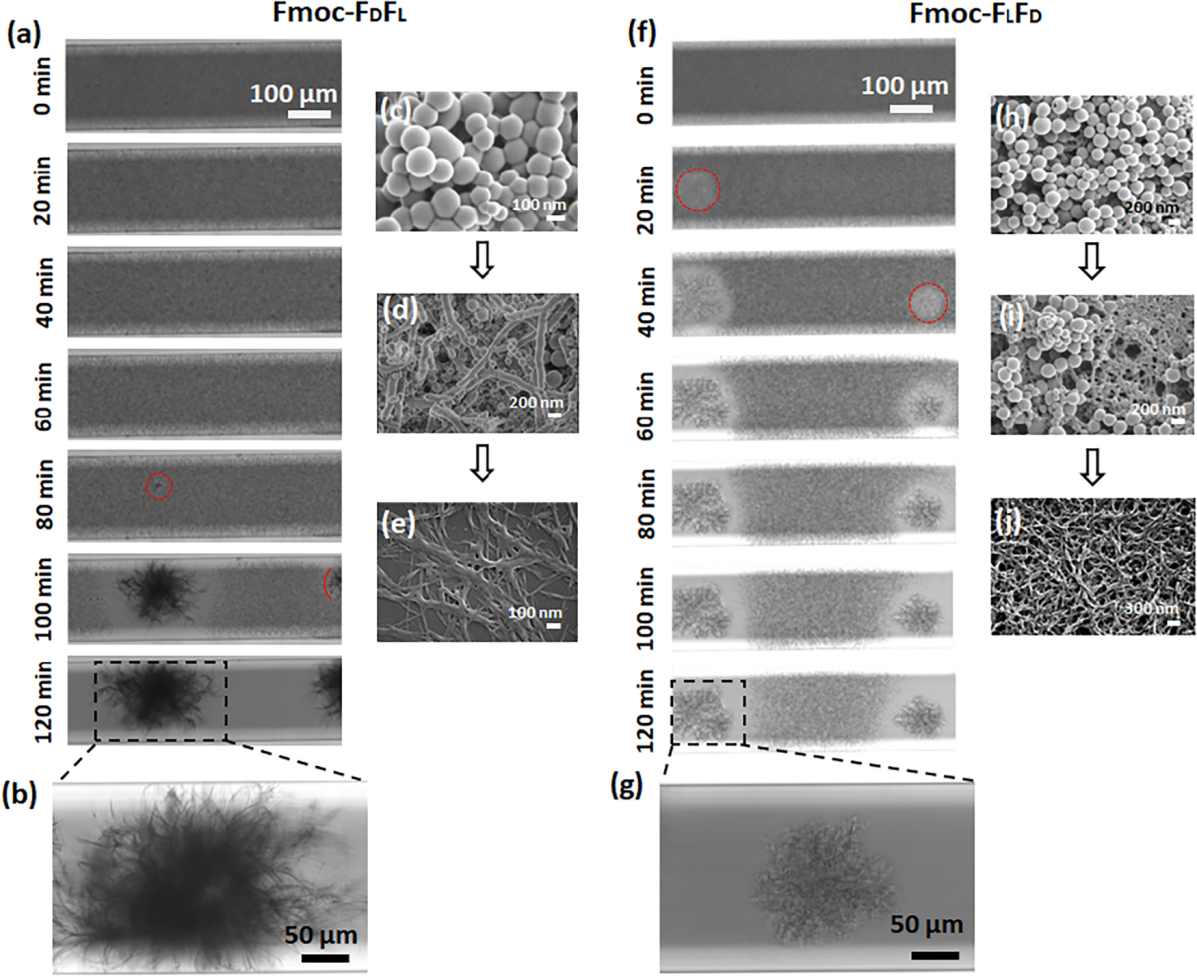
Real-time monitoring of the structural transitions of Fmoc-FF heteroenantiomers. Self-assembly process of (a–e) Fmoc-F_D_F_L_ and (f–j) Fmoc-F_L_F_D_. (a, f) Real-time bright field images. (b, g) Magnified view of the morphology of the fibrillary aggregates. (c–e, h–j) HRSEM images of the self-assembled structures at (c, h) 5 min, (d, i) 90 min, and (e, j) 2 h.

The gelation kinetics of Fmoc-F_L_F_D_ was the slowest among the four peptides (Figure 1f), as was also confirmed by the capillary experiment results (Figure 3f and Movie S4). The morphology of the filamentous aggregates of the two heteroenantiomeric hydrogels was different. A less compact feathery-like morphology was observed for Fmoc-F_D_F_L_, whereas a more compact and dense aggregation was observed for Fmoc-F_L_F_D_ (Figure 3b,g, respectively).

Next, we focused on analyzing the nanometer structures and the phase transition morphologies of the peptide hydrogels during the self-assembly process. For this purpose, the self-assembly process of the peptides (5 mg/mL peptide in 5% DMSO) was arrested at different time points and analyzed using HRSEM, scanning transmission electron microscopy (STEM), and TEM microscopy techniques (Figures 3–5 and S1). The spherical structures were abundant at time points ∼30 s and ∼5 min for the homoenantiomeric and heteroenantiomeric peptides, respectively (Figures 3c,h and S1b,e). HRSEM analysis demonstrated a 3-stage transition from spheres to coalescence of spheres, and finally nanofibers, for the heteroenantiomers at ∼90 min after self-assembly initiation (Figure 3d,i). The HRSEM images revealed the fusion of spheres with each other and their subsequent elongation to transform into nanofibrils (Figures S2 and S3). These stages of phase transition were also analyzed using STEM imaging of Fmoc-F_L_F_D_ (Figure 4), confirming the formation of nanofibers by the conjugation of spheres in garland-like structures. From the nanoscale to the macroscale, the entire assembly process resembled the beads-on-a-string model constituted of spheres in numerous branches spreading from different nucleation points. The average size of the spheres was ∼90 and ∼160 nm for Fmoc-F_D_F_L_ and Fmoc-F_L_F_D_, respectively (Figure 5a,c). Identification of the distinct sphere phase of the homoenantiomers was challenging, as the sphere phase existed for only a few seconds, hindering their measurement. After the complete transformation of spheres into fibrillary structures, the diameter of the stable fibers formed by the homoenantiomers was ∼15 nm (Figures 5e,f and S4), whereas the fiber diameter of the heteroenantiomers was thicker, with an average of ∼22 nm (Figures 5b,d and S4). TEM imaging revealed that Fmoc-F_L_F_L_ displayed a lower degree of nanofiber entanglement, while Fmoc-F_D_F_D_ exhibited the highest degree of nanofiber entanglement. In contrast, the heteroenantiomeric hydrogels displayed nanofibers with an intermediate level of entanglement (Figure 5). Noteworthily, twisted ribbons were observed in the Fmoc-F_D_F_L_ self-assembled hydrogels (Figure 5b).

**Figure 4:**
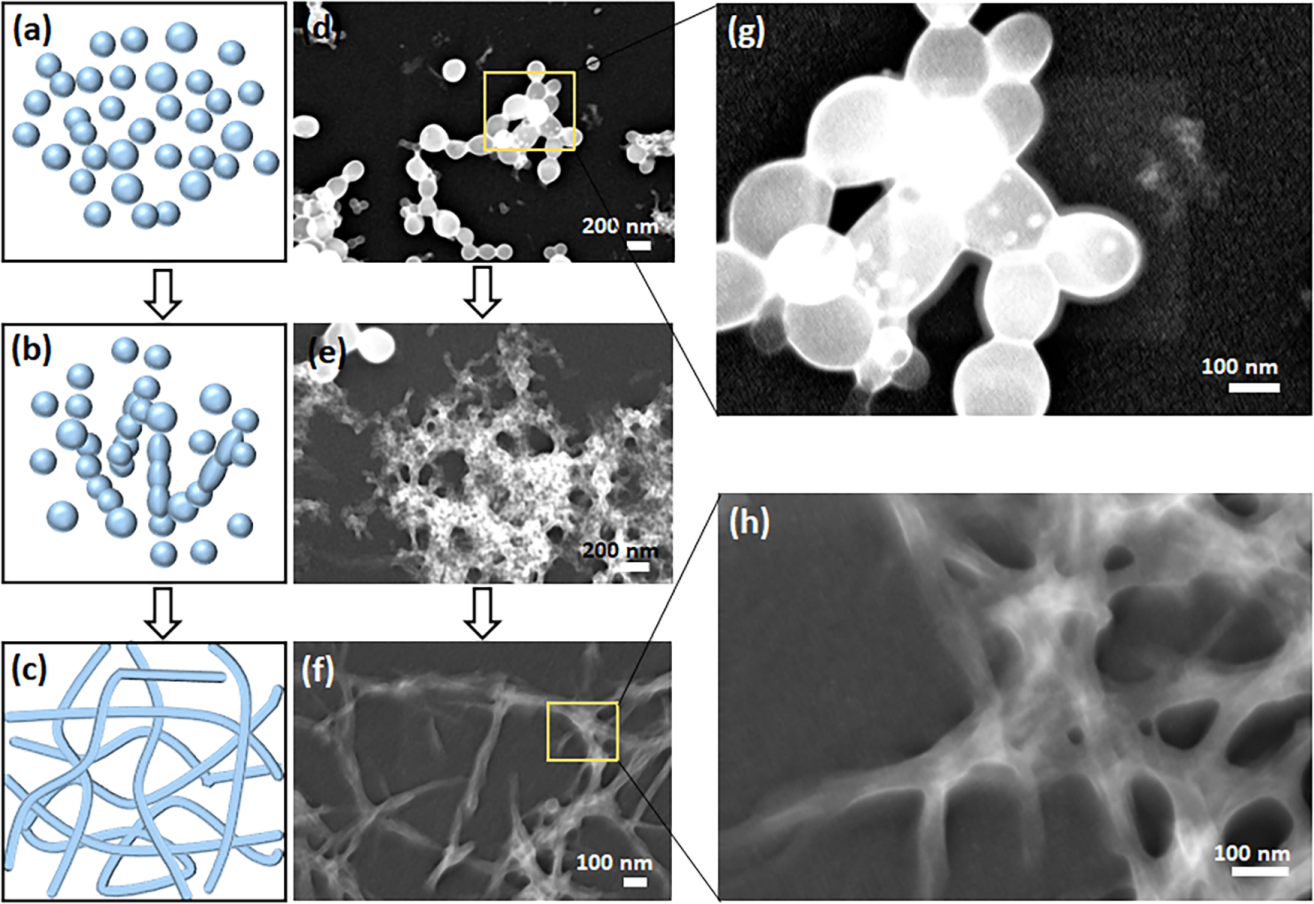
Self-assembly mechanism of the Fmoc-F_L_F_D_ peptide. (a–c) Schematic representation of the self-assembly process from the sphere phase to nanofibrillar structures. (d–f) Corresponding STEM images were taken 90 min after the initiation of the Fmoc-F_L_F_D_ self-assembly process. (g, h) Enlarged view of (g) spheres and (h) fibers.

**Figure 5:**
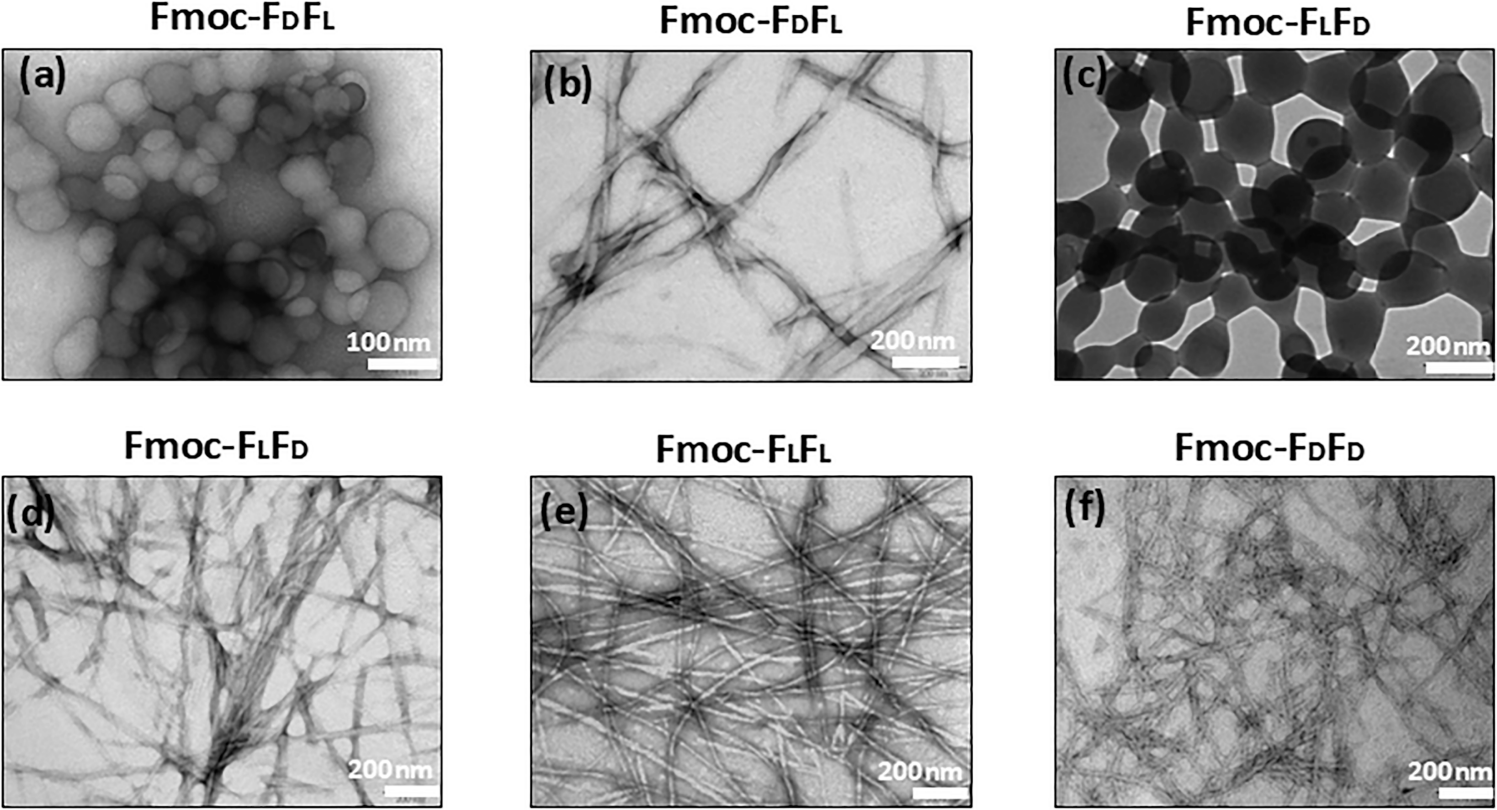
Morphological characterization using TEM. (a) Spherical stage of self-assembly and (b) twisted ribbon of bundled nanofibers of the Fmoc-F_D_F_L_ hydrogel. (c) Spherical stage of self-assembly and (d) bundle of the entangled nanofibers form of the Fmoc-F_L_F_D_ hydrogel. (e) Long and less entangled nanofibers of Fmoc-F_L_F_L_. (f) Bundle of highly entangled nanofibers of Fmoc-F_D_F_D_.

### 2.3 Mechano-Physical Characteristics of the Hydrogels

Rheological analysis was carried out at various temperatures to analyze the different gel formation kinetics and the mechanical properties of the four enantiomeric peptide hydrogels. In this experiment, hydrogels were prepared at 5 mg/mL peptide in 5% DMSO. Oscillatory time sweep measurements were performed at 15, 25, and 45 °C for 4 h at a fixed strain of 0.5% and a frequency of 5 Hz (Figure 6 and Table 1). In agreement with the previous observations, the homoenantiomeric hydrogels rapidly reached a plateau signifying the completion of self-assembled gel formation, compared to the slower process observed for the heteroenantiomeric hydrogels. The self-assembly kinetics and rigidity of the hydrogels were found to be temperature-dependent. With the increase of temperature, a faster self-assembly process was observed, producing a hydrogel with a higher storage modulus. The slowest self-assembly kinetics of each hydrogel was observed at the lowest temperature.

**Figure 6:**
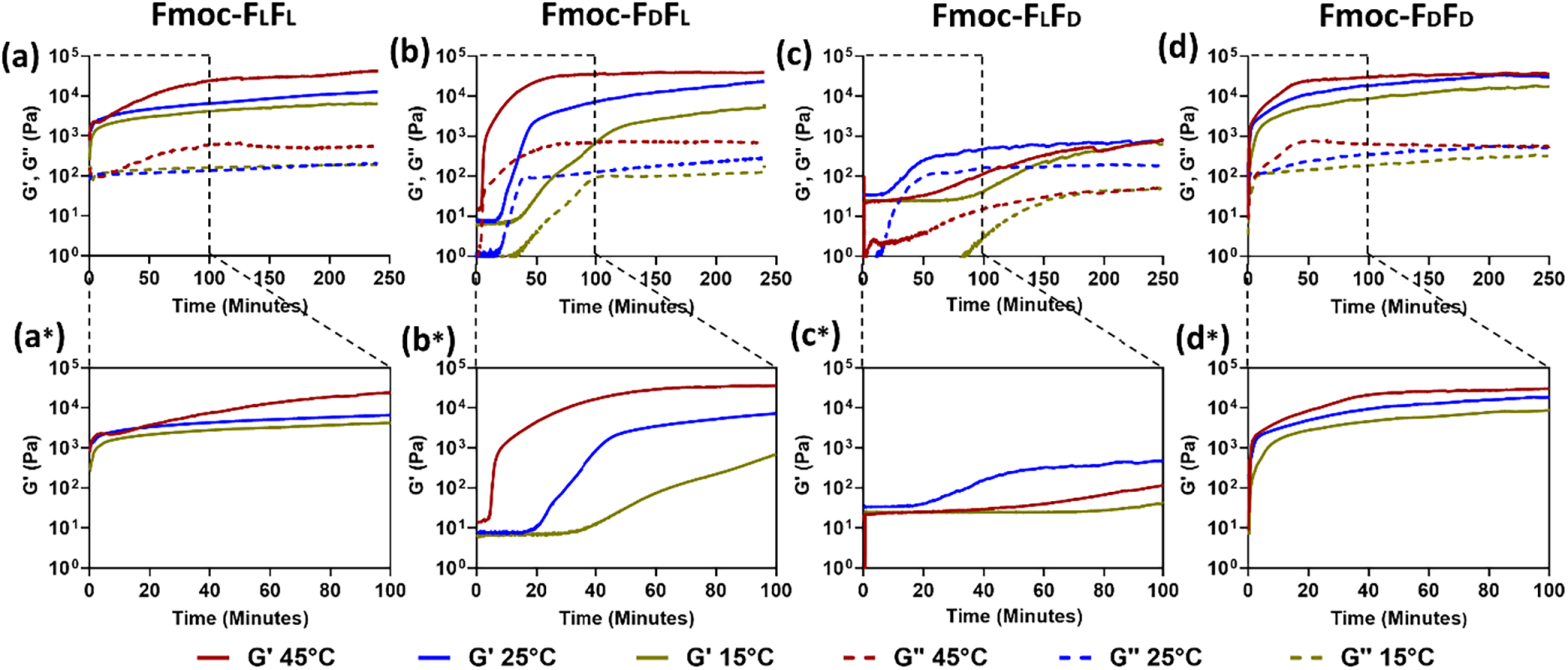
Rheological properties of enantiomeric hydrogels. Time sweep oscillation measurements of storage (*G*′) and loss (*G*″) modulus at different temperatures. (a) Fmoc-F_L_F_L_ and (b) Fmoc-F_D_F_L_. (c) Fmoc-F_L_F_D_. (d) Fmoc-F_D_F_D_. (a*–d*) Corresponding magnified areas of the storage moduli over the initial 100 min, respectively.

**Table 1:** Final Storage Modulus Values (Pa) of the Hydrogels at Different Temperatures

temperature	Fmoc-F_L_F_L_	Fmoc-F_D_F_L_	Fmoc-F_L_F_D_	Fmoc-F_D_F_D_
45 °C	42,829	39,427	779	36,545
25 °C	12,838	23,219	766	30,419
15 °C	6325	5247	635	17,321

Likewise, the hydrogel’s stiffness decreased upon lowering the temperature, with the only exception being Fmoc-F_L_F_D_, which showed a similar storage modulus (*G*′) value at the three different temperatures. Moreover, the elastic modulus (*G*′) was higher than the viscous modulus (*G*″) for the four hydrogels at all temperatures, indicating that the gels were primarily elastic rather than viscous, irrespective of the temperature. As expected, the slowest gelation kinetics and very weak gel properties with a storage modulus of ∼700 Pa at all three temperatures were observed for Fmoc-F_L_F_D_ (Figure 6c,c*). However, the Fmoc-F_D_F_L_ hydrogel showed a high *G*′ value of ∼40 kPa at 45 °C, similar to the homoenantiomeric hydrogels. Although Fmoc-F_D_F_L_ formed a stronger gel than Fmoc-F_L_F_L_ at 25 °C (Figure 6b,b*), the self-assembly kinetics of Fmoc-F_D_F_L_ were slower than the homoenantiomers at all three temperatures until reaching the storage modulus plateau. At 45 °C, the stiffest gel was Fmoc-F_L_F_L_; however, at 25 and 15 °C, the highest storage modulus value of ∼31 and 18 kPa, respectively, was observed for Fmoc-F_D_F_D_ (Table 1). Three different stages were observed in the curve of the heteroenantiomeric gels. Initially, a straight line was detected, then an inclined slope, and finally a plateau was detected (Figure 6b,c). This might be due to the slow self-assembly kinetics of the heteroenantiomers, which allows us to distinctly represent the nucleation stage, self-assembly stage toward an organized structure, and finally stable hydrogel formation. Each stage became longer with the decrease in temperature. Thus, we show that by introducing enantiomer modifications, different self-assembly kinetics and stiffness of the hydrogels can be achieved, which can also be controlled by varying the temperature.

Swelling is an indispensable property of hydrogels, especially for tissue engineering and drug delivery applications.^36,37^ Hydrogels prepared from 5 mg/mL of peptide in 5% DMSO were used to study the swelling ratios. Except for Fmoc-F_L_F_D_, all three other tested hydrogels remained intact in shape with a swollen appearance after overnight incubation in deionized water at 37 °C. In contrast, Fmoc-F_L_F_D_ became fragile during incubation and broke into separate parts. The mean swelling ratios of the Fmoc-F_L_F_L_, Fmoc-F_D_F_L_, and Fmoc-F_D_F_D_ hydrogels were approximately 200, 195, and 183 Ws/Wd, respectively (Figure S5), in contrast to ∼1315 Ws/Wd in the case of Fmoc-F_L_F_D_ (Figure S5). These data are in accordance with the rheological analysis showing the very low storage modulus of Fmoc-F_L_F_D_ (Figure 6c,c*), indicating it is a very weak gel. This difference between the heteroenantiomers may arise from the distinct positioning of the l- and d-amino acids relative to the Fmoc group, which alters the strength and extent of supramolecular interactions, thereby modulating fibril stability and leading to the very weak nature and high swelling ratio of the Fmoc-F_L_F_D_ hydrogel.

### 2.4 Secondary Structure of the Four Enantiomeric Peptides

After exploring the physical properties at the nanoscale, we aimed to determine the secondary structure of the self-assembled hydrogels. The secondary structure conformation of the peptide hydrogels was analyzed by using Fourier-transform infrared (FTIR) spectroscopy (Figure 7a). The characteristic amide-I stretching frequency at ∼1650 cm^–1^ for all hydrogels indicated a β-sheet conformation. A prominent peak at ∼1690 cm^–1^ signifying the carbamate moiety of the Fmoc group was also detected.^38^

**Figure 7:**
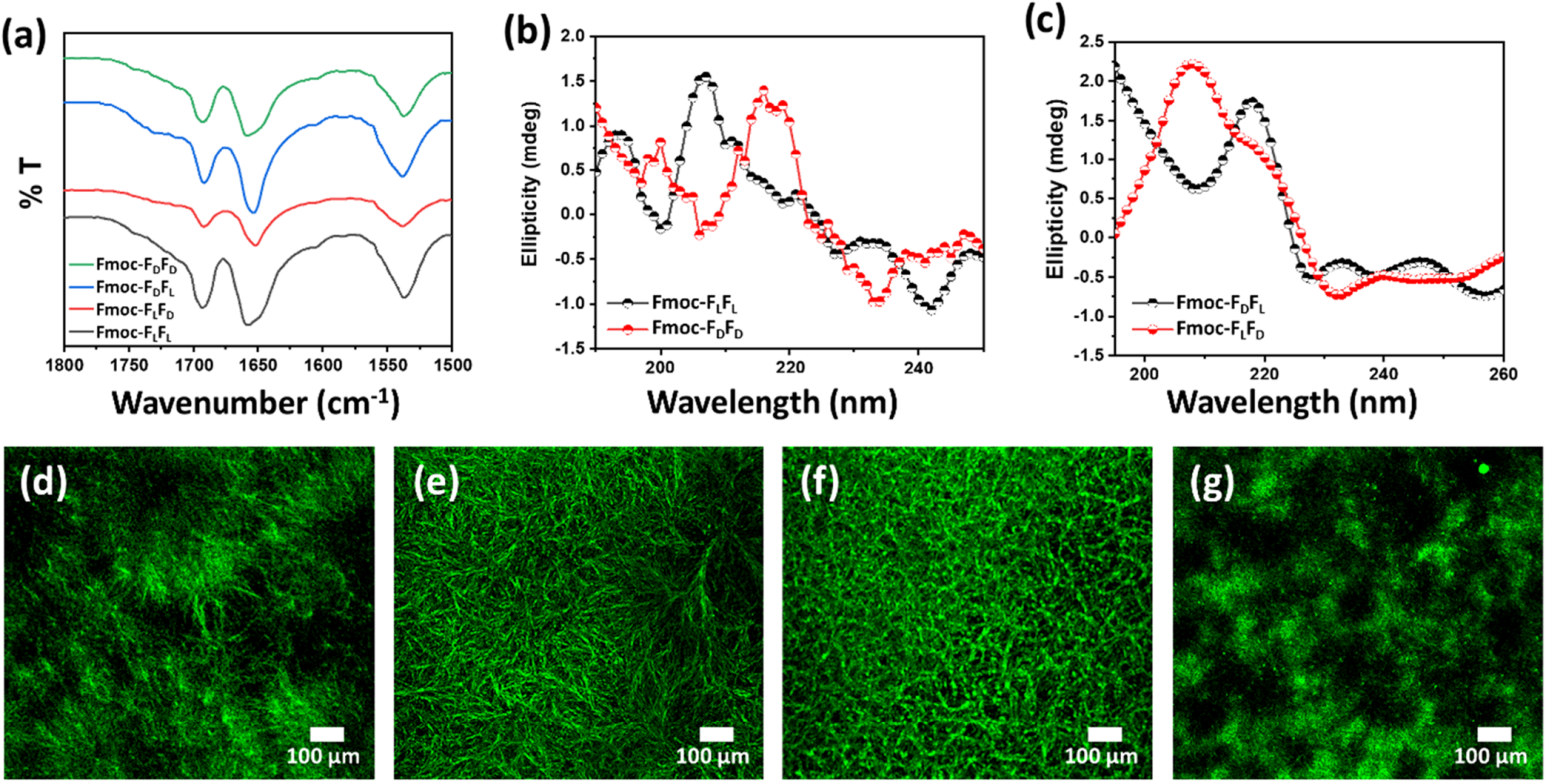
Secondary structure of enantiomeric hydrogels. (a) FTIR spectra. (b, c) Circular dichroism (CD) spectra. (d–g) Confocal microscopy images of the four hydrogel fibers following Thioflavin T (ThT) staining. (d) Fmoc-F_L_F_L_. (e) Fmoc-F_D_F_L_. (f) Fmoc-F_L_F_D_. (g) Fmoc-F_D_F_D_.

The secondary conformation of the hydrogel fibers, synthesized using 5 mg/mL peptide in 5% DMSO, was confirmed by circular dichroism (CD) spectroscopy (Figure 7b,c). The presence of a negative peak at ∼235 nm indicated that all four peptides had adopted a β-sheet conformation during the self-assembly process, similar to the phenomenon reported by Sharma et al.^39^ Fmoc-F_L_F_L_ showed the signature β-sheet peaks, namely, a negative band at ∼218 nm and a positive broad maxima at ∼206 nm, as previously reported (Figure 7b).^40–42^ Likewise, Fmoc-F_D_F_D_ showed a positive peak at ∼200 nm, similar to the Fmoc-F_L_F_L_ β-sheet conformation.^43^ However, a negative peak at ∼233 nm indicated the appearance of a cross-β-sheet-like structure (Figure 7b).^39^ In addition, a negative peak at ∼207 nm and a positive broad maximum in the range of ∼220 nm signified a polyproline II-like characteristic (Figure 7b). A similar polyproline II-like trend was also observed for Fmoc-F_D_F_L_ (Figure 7c). The polyproline II conformation in the secondary structure of Fmoc-dipeptide self-assembly was previously reported by Mu et al. and Fleming et al.^38,44^ In contrast, a strong red shift from the typical β-sheet peak in negative maxima at ∼226 nm with one minimum negative peak and one maximum positive peak emphasized the strongly twisted β-sheet structure of Fmoc-F_D_F_L_ (Figure 7c).^45^ In the case of Fmoc-F_L_F_D_, a broad positive peak at ∼206 nm indicated a β-sheet-like structure, and a negative maximum at ∼233 nm suggested a dominant cross-β-sheet-like conformation (Figure 7c). The difference in the secondary structure of the peptide hydrogel fibers was probably due to variation in the gelation pathways.

The secondary structure of the peptide fibers was further analyzed using a Thioflavin T (ThT) binding assay (Figure 7d–g). ThT is an amyloid-specific fluorescent dye that characteristically binds within the aromatic hydrophobic grooves of the β-sheet on the amyloid surface.^46^ The fluorescence intensity depends on the amyloid microenvironment, increasing by 1000-fold after binding to the amyloidogenic fibers.^47^ The fiber morphology was analyzed by using confocal imaging of ThT-treated hydrogels, showing four distinct morphologies (Figure 7). A completely organized fibril network with separately identifiable ThT-labeled fibers showing high fluorescence intensity was observed for the heteroenantiomeric hydrogels, each with a distinct morphology (Figure 7e,f). However, the fluorescently labeled fibers of the homoenantiomeric hydrogels were bundled (Figure 7d,g). The reason might be the fast self-assembly kinetics of the homoenantiomeric hydrogels, which hinders a uniform distribution of the ThT molecules, whereas the slow self-assembly kinetics of the heteroenantiomeric hydrogels allows ThT molecules to uniformly attach at the specific binding sites in the fibers.

### 2.5 Hydrogel Affinity to Molecular Oxygen

Molecular oxygen (O_2_) significantly inhibits various enzymatic reactions in biological and industrial processes, as it is a very reactive oxidizing agent. Therefore, there is a need to develop a nontoxic biocompatible system to protect oxygen-sensitive molecules from contact with molecular O_2_. Our previous work compared Fmoc-F_L_F_L_ to various other peptides and nonpeptide gels, and found Fmoc-F_L_F_L_ to be efficient in slowing O_2_ penetration. We have also shown that the Fmoc-F_L_F_L_ hydrogel is an efficient system to protect the O_2_-hypersensitive [FeFe]-hydrogenase enzyme by encapsulating the enzyme within the hydrogel.^12^ Here, we studied the O_2_ penetration ability through the four enantiomeric hydrogels, testing their potential utilization as a protective system for O_2_-sensitive biomolecules. The O_2_ penetration kinetics through the four hydrogels were tested using an optical O_2_ measurement system (Pyroscience FireStingO2). The four hydrogels, prepared at 5 mg/mL peptide in 5% DMSO, and the control solution, consisting of 5% DMSO in water, were prepared in probe vials under anaerobic conditions. After complete gelation, the vials were exposed to atmospheric O_2_ and connected to the O_2_ measurement system, allowing us to determine the O_2_ concentration at the bottom of the vial.^12^ A very fast penetration of the O_2_ was observed in the control vial. O_2_ levels started to increase within minutes after exposure to ambient air and reached 250 μM O_2_ after 18 h (Figure 8).

**Figure 8:**
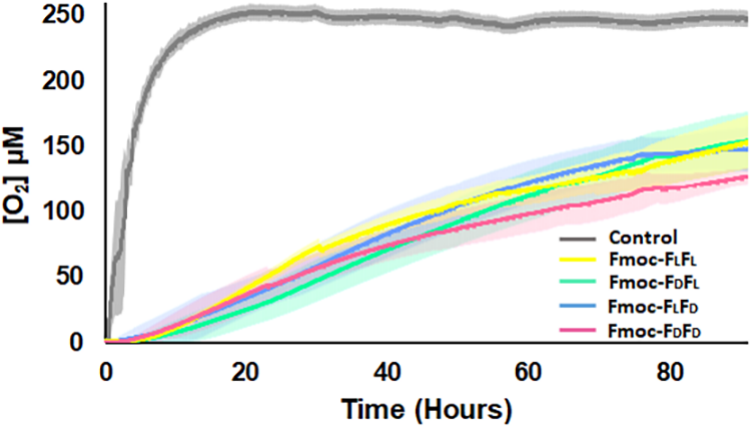
O_2_ penetration through the peptide hydrogels. O_2_ through the four enantiomeric Fmoc-FF hydrogels over time was detected using an optical O_2_ measurement system.

In contrast, very slow penetration of O_2_ through the gels was observed for all four hydrogels. After 18 h, all of the hydrogels remained relatively anoxic with O_2_ concentrations of ∼34, 32, 21, and 29 μM for Fmoc-F_L_F_L_, Fmoc-F_D_F_D_, Fmoc-F_D_F_L_, and Fmoc-F_L_F_D_, respectively (Figure 8). After 88 h, the O_2_ concentration in the Fmoc-F_D_F_D_ hydrogel reached 125 μM, and in all other hydrogels, it reached 150 μM. Therefore, all four enantiomeric gel compositions are efficient in restricting O_2_ penetration through the gel and can be used as a protective medium for O_2_-hypersensitive enzymes in ambient air environments. Noteworthily, the similar attenuated O_2_ penetration throughout all four enantiomers, regardless of the differences in the physical and mechanical properties, could be explained by the formation of hydrophobic pockets, spawned by the supramolecular conformation of Fmoc-FF monomers in the self-assembled fibril structure, which specifically encages O_2_ and limits its diffusion, as previously shown by molecular dynamics.^12^

### 2.6 Enzyme Encapsulation in Heteroenantiomeric Hydrogels

Finally, we encapsulated [FeFe] hydrogenase from *Chlamydomonas reinhardtii* in hydrogels of the two slow-gelating, heteroenantiomeric peptides, namely, FmocF_D_F_L_ and FmocF_L_F_D_, and compared the enzyme’s H_2_-producing activity to its encapsulation in FmocF_L_F_L_. All samples were prepared with 5 mg/mL peptide in 20% DMSO, added to 100 mM tris buffer, pH 7.3. Despite the difference in chirality, we found no significant difference in the activity of this enzyme (Figure 9), suggesting that chirality does not hinder enzyme function, hence enhancing the peptides’ applicability potential.

**Figure 9:**
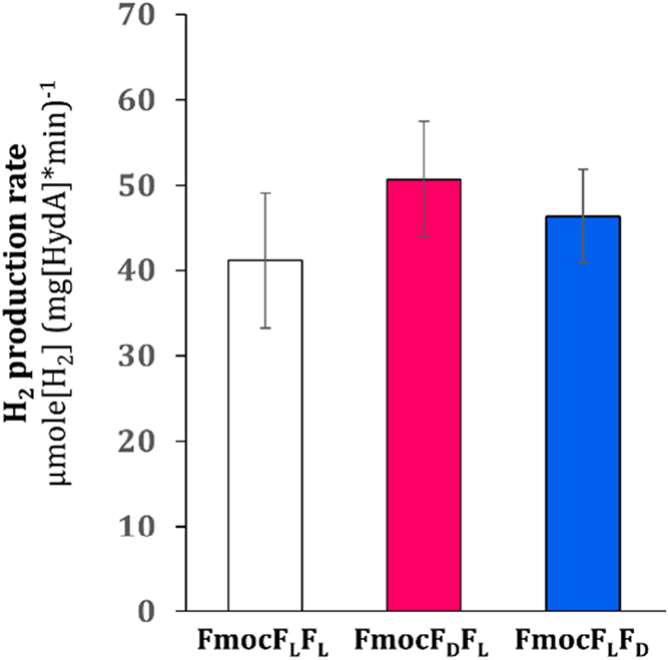
Enzymatic H_2_-production encapsulated in the hydrogels. H_2_-production activity of [FeFe] hydrogenase from *C. reinhardtii*, encapsulated in FmocF_L_F_L_, FmocF_D_F_L_, and FmocF_L_F_D_ hydrogels (*n* = 3).

## 3 Conclusions

This research presents a detailed analysis of the self-assembly kinetics of the different enantiomeric forms of the Fmoc-FF dipeptide. All four enantiomeric combinations of the dipeptide were used to study the difference in assembly kinetics, phase transition, and final hydrogel properties to obtain hydrogels with tunable characteristics. These four enantiomeric hydrogels show promise as an encapsulation system to increase the lifetime of O_2_-hypersensitive molecules. Interestingly, while the homoenantiomeric hydrogels show fast self-assembly kinetics with high storage modulus, the Fmoc-F_D_F_L_ peptide shows intermediate self-assembly kinetics but a storage modulus and swelling ratio similar to the homoenantiomers. In contrast, Fmoc-F_L_F_D_ displays the slowest self-assembly kinetics, a very low storage modulus, and a very high swelling ratio. This difference between the two heteroenantiomers suggests that an equal number and type of chiral residue does not ensure identical behavior, but the specific chirality of each residue in relation to the Fmoc group is also significant. These analyses allowed us to establish that at the beginning of the self-assembly of the heteroenantiomeric peptides, monomers nucleate into nanospheres, which gradually attach and finally transform into stable nanofibers. The rate of transformation differs from one enantiomeric form to another. For the four different enantiomeric hydrogels, different fiber morphologies and secondary peptide structures were observed. It can be inferred that the enantiomeric arrangement of amino acids in the monomer peptides has a significant effect on the self-assembly kinetics, leading to the attainment of specific mechanical properties and nanostructured morphologies of 3D hydrogel materials. This can be an advantageous tool for various tissue engineering applications. For instance, the mechanical properties of the cell’s surrounding environment play a crucial role in regulating stem cell differentiation,^48^ making it a notable example of how this stiffness variation can be leveraged. Moreover, slower gelation may be highly advantageous for controlled encapsulation, as it can promote a more homogeneous distribution of the cargo, particularly in systems with limited volume or numerous nucleation centers. A slowed gelation can also improve the ease of handling and sample preparation, facilitating applications requiring injection or molding into defined shapes. The advantage of heteroenantiomers, when encapsulation in a slow-gelating medium is desired, is further highlighted by the comparable activity of the H_2_-producing enzyme in both homo- and heteroenantiomeric hydrogels.

## 4 Materials and Methods

### 4.1 Materials

All of the peptides were purchased from GL Biochem (Shanghai) Ltd., China, with purification >95%, confirmed by HPLC. Peptides were kept at −20 °C. Dimethyl sulfoxide (DMSO) and Thioflavin T (ThT) were purchased from Sigma-Aldrich (Rehovot, Israel).

### 4.2 Hydrogel Synthesis

Peptide hydrogels were prepared as previously described.^30,33,49^ Briefly, four types of peptide stock solutions (100 mg/mL) were prepared in DMSO. The hydrogel was self-assembled by using the solvent switch method. For all experiments except phase characteristics and real-time monitoring of the self-assembly process, 5 mg/mL of peptide and 5% DMSO were used. For these two experiments, a certain amount of peptide stock solution was mixed with double-distilled water (DDW) to obtain the desired peptide concentration and DMSO ratio in the final hydrogel. The mixture was incubated at room temperature until the formation of a hydrogel.

### 4.3 Morphological Characterization of Phase Transition

#### 4.3.1 Optical Microscopy

An array of peptide concentrations (0.2–10 mg/mL) versus a range of DMSO ratios (10–100%) was prepared in 96-well plates and equilibrated for 24 h. The plates were studied to analyze the state of gel formation and the morphological differences of the gel depending on the peptide concentration and DMSO ratios. The ordered structure was monitored under an optical microscope every day for 7 days and followed up every week.

#### 4.3.2 Real-Time Monitoring of the Self-Assembly Process

Hydrogels of the four Fmoc-FF enantiomeric peptides (10 mg/mL peptide in 60% DMSO) were prepared as outlined above (Section 4.2), and the turbid suspension was immediately transferred into square glass capillaries. Both open ends of each capillary were sealed to prevent evaporation, and the self-assembly process was monitored by time-lapse imaging using an optical microscope.

#### 4.3.3 Transmission Electron Microscopy (TEM)

Hydrogels were prepared using 5 mg/mL of peptide in 5% DMSO. 10 μL of each hydrogel was applied to 400-mesh copper grids (Electron Microscopy Sciences, Ltd.), and the excess liquid was removed after 1 min. The assembly was arrested at different time points by drying the hydrogel on a copper grid. The samples were negatively stained by 10 μL of 2% uranyl acetate for 1 min and imaged using a JEOL JEM-2010F TEM (Japan).

#### 4.3.4 High-Resolution Scanning Electron Microscopy (HRSEM)

A 10 μL drop of each hydrogel, synthesized at 5 mg/mL peptide in 5% DMSO, was applied to a copper grid, followed by elimination of excess liquid to arrest the self-assembly process at different time points. The time points were determined based on the real-time self-assembly experiment in a glass capillary. Following the initiation of self-assembly, the excess liquid was removed after 5, 90, and 120 min for Fmoc-F_D_F_L_, after 5, 90, and 240 min for Fmoc-F_L_F_D_, and 30 s, 5 min for Fmoc-F_L_F_L_ and Fmoc-F_D_F_D_. All of the grids were coated with a 6 nm thick film of gold before analysis using Zeiss GeminiSEM 300 HRSEM.

### 4.4 Rheological Analysis

Temperature-dependent self-assembly kinetics and mechanical properties of the four enantiomeric hydrogels (5 mg/mL peptide in 5% DMSO) were studied using an AR-G2 rheometer (TA Instruments). Oscillatory strain (0.01–100%) and frequency sweep (0.01–100 Hz) measurements were conducted to determine the linear viscoelastic region. This allowed optimization of the parameters for performing the time sweep oscillatory tests of the instantly prepared hydrogels. The storage (*G*′) and loss (*G*″) moduli were determined at different temperatures (45, 25, and 15 °C) for 4 h at a constant frequency (5 Hz) and strain (0.5%).

### 4.5 Secondary Structure Characterization

#### 4.5.1 Fourier-Transform Infrared (FTIR) Spectroscopy

Peptide hydrogels (5 mg/mL peptide in 5% DMSO) were drop-cast on KBr infrared cards (Sigma-Aldrich) and completely dried under a vacuum. Samples were saturated twice with D_2_O and dried in a vacuum to eliminate the water signal. Then, FTIR analysis was performed using a nitrogen-purged Nicolet Nexus 470 FTIR spectrometer (Nicolet, Offenbach, Germany) equipped with a deuterated triglycine sulfate detector, as previously described.^31^

#### 4.5.2 Circular Dichroism (CD) Spectroscopy

The secondary structure of the self-assembled hydrogels (5 mg/mL peptide in 5% DMSO) formed by the four Fmoc-FF enantiomeric peptides was analyzed using a Chirascan spectrometer (Applied Photophysics, Leatherhead, U.K.). A Peltier temperature controller set to 25 °C was attached to the spectrometer, and quartz cuvettes with an optical path length of 0.1 mm (Hellma Analytics) were used. Absorbance of the sample was kept within the linear range of the instrument during the measurements. Data was acquired within the wavelength range of 195–260 at 1 nm intervals. The bandwidth of the spectrum was fixed at 1.0 nm, and each measurement was averaged over 3 s. The spectrum of each sample was collected three times and averaged. Data processing was performed by using the Pro-Data Viewer software (Applied Photophysics).

#### 4.5.3 Thioflavin T (ThT) Binding Assay

The peptide stock solution (100 mg/mL) was diluted in ThT-containing aqueous solution (0.075 mg/mL) in order to prepare the 5 mg/mL peptides in 5% DMSO. After complete gelation, the hydrogels were examined under a confocal microscope (ZEISS LSM 900, ZEISS Germany) with an excitation at 440 nm and an emission at 478 nm.

### 4.6 Swelling Assay

Equal volumes of 1 mL of the four hydrogels (5 mg/mL in 5% DMSO) were transferred to Petri dishes, each with four identical repeats. The initial weights (Wi) of the hydrogels were recorded, and the hydrogels were incubated for 24 h in DDW at 37 °C. The swollen mass (Ws) was measured by removing the DDW and eventually lyophilizing the hydrogels to obtain the dry weight (Wd). The hydrogels’ swelling ratio was calculated by dividing Ws by Wd.^30^

### 4.7 Encapsulated Enzyme Activity Assay

Enzymatic activity of [FeFe] hydrogenase from *C. reinhardtii* was tested using a previously reported H_2_ production assay, adapted to assess the enzyme encapsulated within peptide hydrogels.^12^ Gels were prepared by dissolving 5 mg/mL Fmoc-FF enantiomers in 20% DMSO and diluting them with 100 mM tris buffer at pH 7.3 to initiate self-assembly. Sodium dithionite and methyl viologen were used as the electron donor and mediator, respectively, and H_2_ production was quantified via gas chromatography.

### 4.8 O_2_ Penetration Assay

Hydrogels (5 mg/mL peptide in 5% DMSO) were prepared in 1 mM sodium dithionite and 1 mM methyl viologen (MV) containing 100 mM Tris-HCl buffer (pH 7.2) in 4 mL OXVIAL4 oxygen sensor vials (PyroScience, Germany) in an anaerobic chamber. After complete gelation, the vials were sealed and removed from the chamber. A vial without any peptide was used as a control. The sensors of the vials, FireStingO2 FSO2–4, and a temperature meter (PyroScience, Germany) were connected through an optical fiber. The vials were then opened in ambient air, and O_2_ concentration was measured continuously. Measurement was normalized according to ambient temperature and pressure.

## Supplementary Material











## Notes

Additional experimental results: real-time monitoring of the structural transitions of the homoenantiomers (Figure S1); HRSEM imaging of the self-assembly process of the Fmoc-F_D_F_L_ peptide (Figure S2); HRSEM imaging of the self-assembly process of the Fmoc-F_L_F_D_ peptide (Figure S3); box plot analysis of nanostructure diameters measured based on TEM images of the four Fmoc-FF enantiomeric hydrogels (Figure S4); and swelling ratio of four peptide hydrogels (Figure S5) (PDF)
Real-time monitoring of the structural transitions of Fmoc-F_L_F_L_ (Movie S1) (MP4)
Real-time monitoring of the structural transitions of Fmoc-F_D_F_D_ (Movie S2) (MP4)
Real-time monitoring of the structural transitions of Fmoc-F_D_F_L_ (Movie S3) (MP4)
Real-time monitoring of the structural transitions of Fmoc-F_L_F_D_ (Movie S4) (MP4)

## References

[1] De
Santis E., Ryadnov M. G. (2015). Peptide self-assembly for nanomaterials:
The old new kid on the block. Chem. Soc. Rev..

[2] Simonson A. W., Aronson M. R., Medina S. H. (2020). Supramolecular
peptide assemblies
as antimicrobial scaffolds. Molecules.

[3] Okesola B. O., Wu Y., Derkus B., Gani S., Wu D., Knani D., Smith D. K., Adams D. J., Mata A. (2019). Supramolecular Self-Assembly
to Control Structural and Biological Properties of Multicomponent
Hydrogels. Chem. Mater..

[4] Lee S., Trinh T. H. T., Yoo M., Shin J., Lee H., Kim J., Hwang E., Lim Y. B., Ryou C. (2019). Self-assembling peptides
and their application in the treatment of diseases. Int. J. Mol. Sci..

[5] Majkowska A., Inostroza-Brito K. E., Gonzalez M., Redondo-Gómez C., Rice A., Rodriguez-Cabello J.
C., Del Rio Hernandez A. E., Mata A. (2023). Peptide-Protein Coassemblies into Hierarchical and Bioactive Tubular
Membranes. Biomacromolecules.

[6] Li L., Xie L., Zheng R., Sun R. (2021). Self-Assembly Dipeptide
Hydrogel:
The Structures and Properties. Front. Chem..

[7] Reches M., Gazit E. (2003). Casting metal nanowires
within discrete self-assembled peptide nanotubes. Science.

[8] Diaferia C., Rosa E., Accardo A., Morelli G. (2021). Peptide-based hydrogels
as delivery systems for doxorubicin. J. Pept.
Sci..

[9] Frederix P. W. J. M., Ulijn R. V., Hunt N. T., Tuttle T. (2011). Virtual screening for
dipeptide aggregation: Toward predictive tools for peptide self-Assembly. J. Phys. Chem. Lett..

[10] Wang Y., Geng Q., Zhang Y., Adler-Abramovich L., Fan X., Mei D., Gazit E., Tao K. (2023). Fmoc-diphenylalanine
gelating nanoarchitectonics: A simplistic peptide self-assembly to
meet complex applications. J. Colloid Interface
Sci..

[11] Tao K., Levin A., Adler-Abramovichab L., Gazit E. (2016). Fmoc-Modified Amino
Acids and Short Peptides: Simple Bio- Inspired Building Blocks for
the Fabrication of Functional Materials. Chem.
Soc. Rev..

[12] Ben-Zvi O., Grinberg I., Orr A. A., Noy D., Tamamis P., Yacoby I., Adler-Abramovich L. (2021). Protection
of oxygen-sensitive enzymes
by peptide hydrogel. ACS Nano.

[13] Yang X., Ma L., Lu K., Zhao D. (2024). Mechanism of Peptide Self-assembly
and Its Study in Biomedicine. Protein J..

[14] Zheng Y., Mao K., Chen S., Zhu H. (2021). Chirality Effects in Peptide Assembly
Structures. Front. Bioeng. Biotechnol..

[15] Zheng H., Yoshitomi T., Yoshimoto K. (2018). Analysis of chirality effects on
stem cell fate using threedimensional fibrous peptide hydrogels. ACS Appl. Bio Mater..

[16] Dubovski, N. ; Shoshan-Galeczki, Y. B. ; Malach, E. ; Niv, M. Y. Sweet chirality: The taste of L: The D-glucose stereoisomers bioRxiv 2020 10.1101/2020.08.16.252718.

[17] Marchesan S., Easton C. D., Kushkaki F., Waddington L., Hartley P. G. (2012). Tripeptide self-assembled hydrogels: Unexpected twists
of chirality. Chem. Commun..

[18] Arakawa H., Takeda K., Higashi S. L., Shibata A., Kitamura Y., Ikeda M. (2020). Self-assembly and hydrogel
formation ability of Fmoc-dipeptides comprising
α-methyl-L-phenylalanine. Polym. J..

[19] Zhang G., Zhang L., Rao H., Wang Y., Li Q., Qi W., Yang X., Su R., He Z. (2020). Role of molecular chirality
and solvents in directing the self-assembly of peptide into an ultra-pH-sensitive
hydrogel. J. Colloid Interface Sci..

[20] Garcia A. M., Melchionna M., Bellotto O., Kralj S., Semeraro S., Parisi E., Iglesias D., D’Andrea P., De Zorzi R., Vargiu A. V., Marchesan S. (2021). Nanoscale
assembly of functional peptides with divergent programming elements. ACS Nano.

[21] McAulay K., Dietrich B., Su H., Scott M. T., Rogers S., Al-Hilaly Y. K., Cui H., Serpell L. C., Seddon A. M., Draper E. R., Adams D. J. (2019). Using chirality to influence supramolecular
gelation. Chem. Sci..

[22] Aviv M., Cohen-Gerassi D., Orr A. A., Misra R., Arnon Z. A., Shimon L. J. W., Shacham-Diamand Y., Tamamis P., Adler-Abramovich L. (2021). Modification
of a single atom affects the physical properties of double fluorinated
Fmoc-Phe derivatives. Int. J. Mol. Sci..

[23] Castro V. I. B., Araújo A. R., Reis R. L., Pashkuleva I., Pires R. A. (2025). Nanoengineered self-assembling
peptides with increased
proteolytic stability promote wound healing. ACS Appl. Mater. Interfaces.

[24] Giordano S., Gallo E., Diaferia C., Rosa E., Carrese B., Borbone N., Scognamiglio P. L., Franzese M., Oliviero G., Accardo A. (2023). Multicomponent peptide-based hydrogels containing chemical
functional groups as innovative platforms for biotechnological applications. Gels.

[25] Levin A., Hakala T. A., Schnaider L., Bernardes G. J. L., Gazit E., Knowles T. P. J. (2020). Biomimetic peptide self-assembly
for functional materials. Nat. Rev. Chem..

[26] Zelenovskiy P. S., Nuraeva A. S., Kopyl S., Arkhipov S. G., Vasilev S. G., Bystrov V. S., Gruzdev D. A., Waliczek M., Svitlyk V., Shur V. Y., Mafra L., Kholkin A. L. (2019). Chirality-Dependent
Growth of Self-Assembled Diphenylalanine Microtubes. Cryst. Growth Des..

[27] Kralj S., Bellotto O., Parisi E., Garcia A. M., Iglesias D., Semeraro S., Deganutti C., D’Andrea P., Vargiu A. V., Geremia S., De Zorzi R., Marchesan S. (2020). Heterochirality
and Halogenation Control Phe-Phe Hierarchical Assembly. ACS Nano.

[28] Saile L., Dai K., Pol M. D., Pramod T., Thomann R., Pappas C. G. (2025). Chirality
Makes or Breaks Chemically Driven Self-Assembly. Angew. Chem., Int. Ed..

[29] Bera S., Xue B., Rehak P., Jacoby G., Ji W., Shimon L. J. W., Beck R., Král P., Cao Y., Gazit E. (2020). Self-Assembly
of Aromatic Amino Acid Enantiomers into Supramolecular Materials of
High Rigidity. ACS Nano.

[30] Aviv M., Halperin-Sternfeld M., Grigoriants I., Buzhansky L., Mironi-Harpaz I., Seliktar D., Einav S., Nevo Z., Adler-Abramovich L. (2018). Improving
the mechanical rigidity of hyaluronic acid
by integration of a supramolecular peptide matrix. ACS Appl. Mater. Interfaces.

[31] Bera S., Cazade P.-A., Bhattacharya S., Guerin S., Ghosh M., Netti F., Thompson D., Adler-Abramovich L. (2022). Molecular
engineering of rigid hydrogels co-assembled from collagenous helical
peptides based on a single triplet motif. ACS
Appl. Mater. Interfaces.

[32] Fichman G., Guterman T., Damron J., Adler-Abramovich L., Schmidt J., Kesselman E., Shimon L. J. W., Ramamoorthy A., Talmon Y., Gazit E. (2016). Supramolecular Chemistry: Spontaneous
structural transition and crystal formation in minimal supramolecular
polymer model. Sci. Adv..

[33] Cohen-Gerassi D., Arnon Z. A., Guterman T., Levin A., Ghosh M., Aviv M., Levy D., Knowles T. P. J., Shacham-Diamand Y., Adler-Abramovich L. (2020). Phase Transition
and Crystallization Kinetics of a
Supramolecular System in a Microfluidic Platform. Chem. Mater..

[34] Levin A., Mason T. O., Adler-Abramovich L., Buell A. K., Meisl G., Galvagnion C., Bram Y., Stratford S. A., Dobson C. M., Knowles T. P. J., Gazit E. (2014). Ostwald’s rule
of stages governs structural transitions and morphology of dipeptide
supramolecular polymers. Nat. Commun..

[35] Jeong B., Kim S. W., Bae Y. H. (2012). Thermosensitive
sol-gel reversible
hydrogels. Adv. Drug Delivery Rev..

[36] Spicer C. D. (2020). Hydrogel
scaffolds for tissue engineering: The importance of polymer choice. Polym. Chem..

[37] Lei L., Bai Y., Qin X., Liu J., Huang W., Lv Q. (2022). Current Understanding
of Hydrogel for Drug Release and Tissue Engineering. Gels.

[38] Fleming S., Frederix P. W. J. M., Ramos Sasselli I., Hunt N. T., Ulijn R. V., Tuttle T. (2013). Assessing the utility of infrared spectroscopy as a
structural diagnostic tool for β-sheets in self-assembling aromatic
peptide amphiphiles. Langmuir.

[39] Sharma P., Kaur H., Roy S. (2019). Designing
a Tenascin-C-Inspired Short
Bioactive Peptide Scaffold to Direct and Control Cellular Behavior. ACS Biomater. Sci. Eng..

[40] Feng Z., Su Q., Zhang C., Huang P., Song H., Dong A., Kong D., Wang W. (2020). Bioinspired Nanofibrous Glycopeptide
Hydrogel Dressing for Accelerating Wound Healing: A Cytokine-Free,
M2-Type Macrophage Polarization Approach. Adv.
Funct. Mater..

[41] Elsawy M. A., Smith A. M., Hodson N., Squires A., Miller A. F., Saiani A. (2016). Modification of β-Sheet
Forming Peptide Hydrophobic
Face: Effect on Self-Assembly and Gelation. Langmuir.

[42] Li J., Du X., Hashim S., Shy A., Xu B. (2017). Aromatic-aromatic interactions
enable α-helix to β-sheet transition of peptides to form
supramolecular hydrogels. J. Am. Chem. Soc..

[43] Corrêa D., Ramos C. (2009). The use of circular
dichroism spectroscopy to study protein folding,
form and function. Afr. J. Biochem. Res..

[44] Mu X., Eckes K. M., Nguyen M. M., Suggs L. J., Ren P. (2012). Experimental
and computational studies reveal an alternative supramolecular structure
for Fmoc-dipeptide self-assembly. Biomacromolecules.

[45] Ji W., Yuan C., Chakraborty P., Gilead S., Yan X., Gazit E. (2019). Stoichiometry-controlled
secondary structure transition of amyloid-derived
supramolecular dipeptide co-assemblies. Commun.
Chem..

[46] Ziaunys M., Sakalauskas A., Smirnovas V. (2020). Identifying Insulin Fibril Conformational
Differences by Thioflavin-T Binding Characteristics. Biomacromolecules.

[47] Tikhonova T. N., Rovnyagina N. N., Arnon Z. A., Yakimov B. P., Efremov Y. M., Cohen-Gerassi D., Halperin-Sternfeld M., Kosheleva N. V., Drachev V. P., Svistunov A. A., Timashev P. S., Adler-Abramovich L., Shirshin E. A. (2021). Mechanical Enhancement
and Kinetics Regulation of Fmoc-Diphenylalanine
Hydrogels by Thioflavin T. Angew. Chem..

[48] Vining K. H., Mooney D. J. (2017). Mechanical forces
direct stem cell behaviour in development
and regeneration. Nat. Rev. Mol. Cell Biol..

[49] Schnaider L., Ghosh M., Bychenko D., Grigoriants I., Ya’ari S., Shalev Antsel T., Matalon S., Sarig R., Brosh T., Pilo R., Gazit E., Adler-Abramovich L. (2019). Enhanced nanoassembly-incorporated
antibacterial composite materials. ACS Appl.
Mater. Interfaces.

