# Supplementary material for: Controlling Supramolecular Assembly through Peptide Chirality
Source: ACS Appl Mater Interfaces. 2025 Nov 25;17(49):66998–7009. doi: 10.1021/acsami.5c14913 (PMC12874373; doi:10.1021/acsami.5c14913)
Supplement: Supplementary file 1 [file am5c14913_si_001.pdf]

Supporting Information

Controlling Supramolecular Assembly through Peptide Chirality

Manosree Chatterjee[1,2,3‡], *Itzhak Grinberg*[1,2,3,4‡], *Santu Bera*[1,2,3], *Dana Cohen-Gerassi*[1,2,3,5], *Oren Ben-Zvi*[4,6], *Iftach Yacoby*[4,6], *Moran Aviv*[1,7], *and Lihi Adler-Abramovich*[1,2,3,4*]

[1] Department of Oral Biology, The Goldschleger School of Dental Medicine, The Gray Faculty of Medical and Health Sciences, Tel Aviv University, Tel Aviv 6997801, Israel
[2]The Jan Koum Center for Nanoscience and Nanotechnology, Tel Aviv University, Tel Aviv 6997801, Israel
[3]The Center for the Physics and Chemistry of Living Systems, Tel Aviv University, Tel Aviv 6997801, Israel
[4] New Environmental School (NES), Tel Aviv University, Tel Aviv 6997801, Israel
[5] Department of Materials Science and Engineering, Tel Aviv University, Tel Aviv 6997801, Israel
[6] School of Plant Sciences and Food Security, The George S. Wise Faculty of Life Sciences, Tel Aviv University, Tel Aviv 6997801, Israel
[7] School of Mechanical Engineering, Afeka Tel Aviv Academic College of Engineering, Tel Aviv 6910717, Israel

‡Author Contributions: Manosree Chatterjee and Itzhak Grinberg contributed equally to this work.

*Corresponding Authors E-mail: Lihia@tauex.tau.ac.il (L.A.-A.)

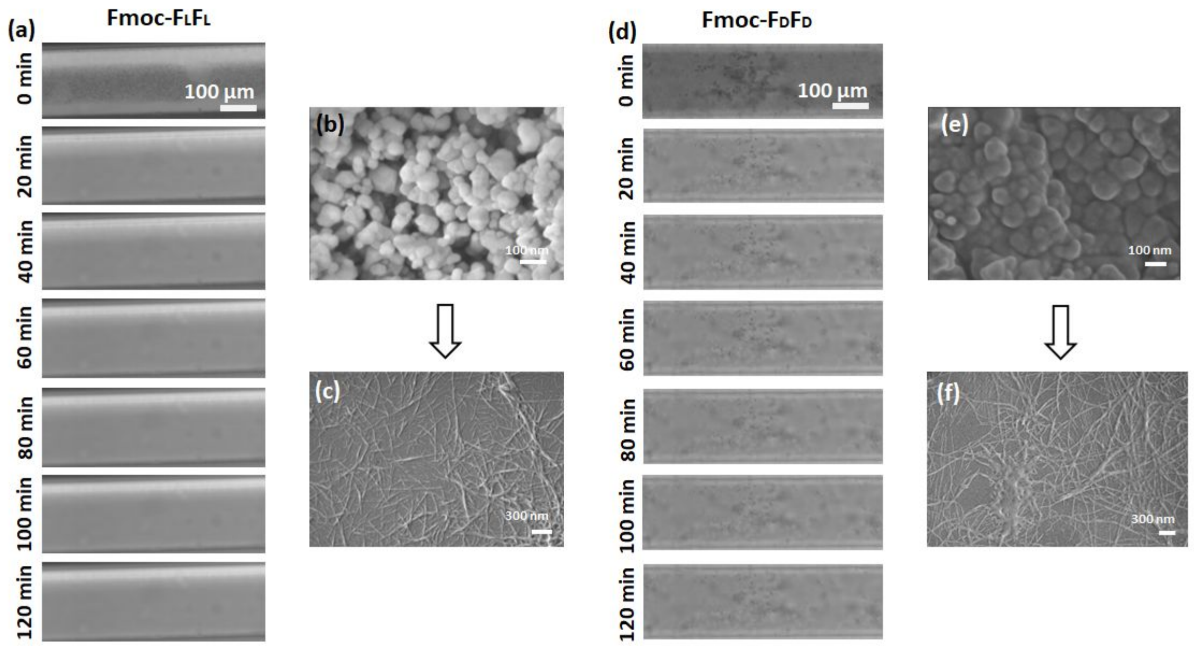

Figure S1. Real-time monitoring of the structural transitions of the homo-enantiomers. (a and d) Bright field observation of real-time self-assembly process of 10 mg/mL (a) Fmoc-F_LF_L and (d) Fmoc-F_DF_D peptides in 60% DMSO in glass capillaries at different time points. (b, c, e, f) HRSEM images of the self-assembly process of (b, c) Fmoc-F_LF_L and (e, f) Fmoc-F_DF_D at different time points after self-assembly initiation: (b and e) 30 seconds, and (c and f) 5 minutes.

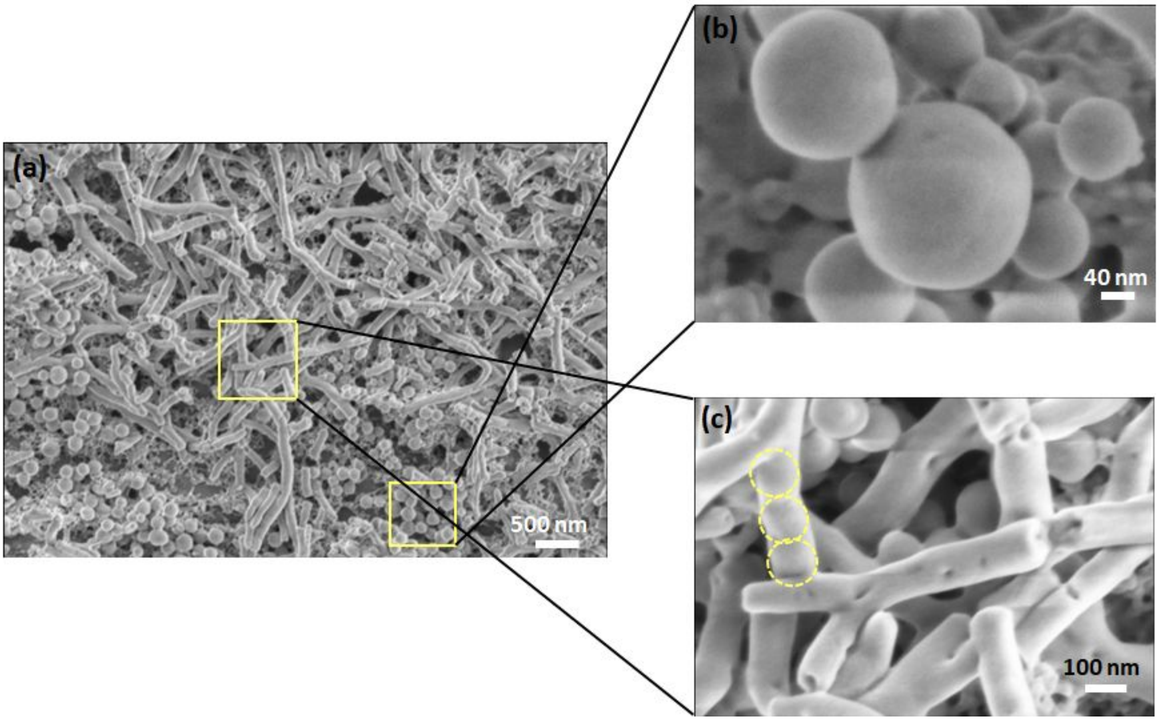

Figure S2. HRSEM imaging of the self-assembly process of the Fmoc-F_DF_L peptide. (a) The sample was prepared by halting the self-assembly at 90 minutes. (b) Magnified view of the spheres. (c) Magnified view of the transition to nano-fiber by the fusion of spheres.

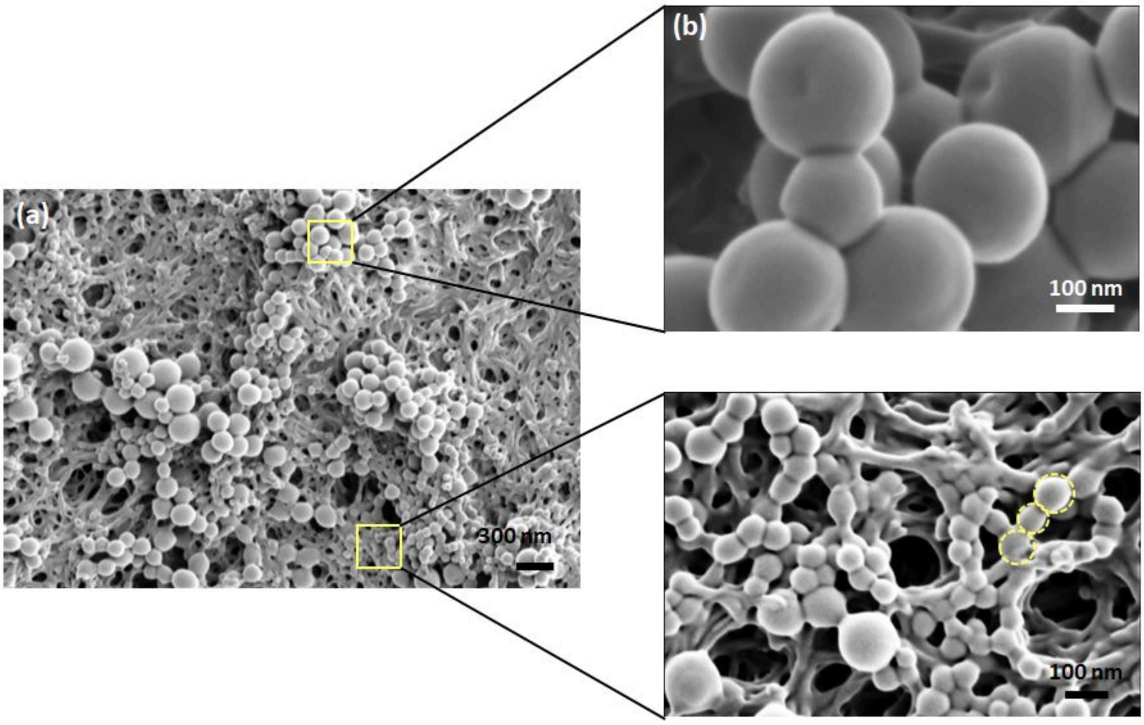

Figure S3. HRSEM imaging of the self-assembly process of the Fmoc-F_LF_D peptide. (a) The sample was prepared by halting the self-assembly at 30 minutes. (b) Magnified view of the spheres. (c) Magnified view of the transition to nano-fiber by the fusion of spheres.

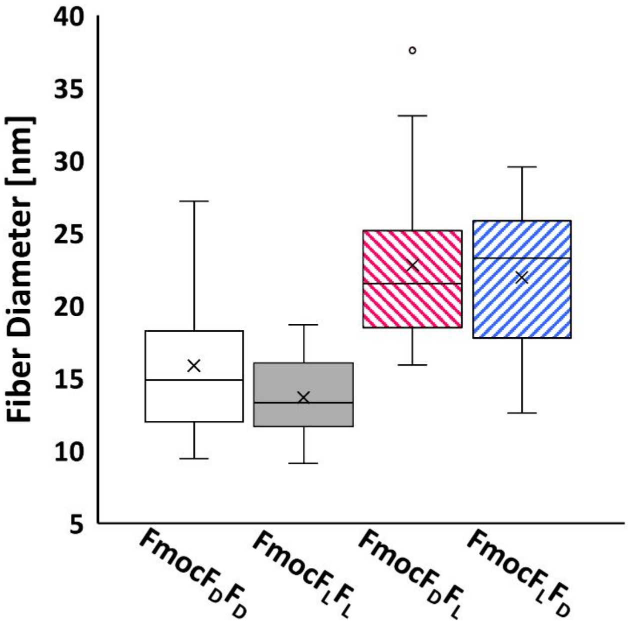

Figure S4. Box plot analysis of nanostructure diameters measured based on TEM images of the four Fmoc-FF enantiomeric hydrogels ($n \geq 29$).

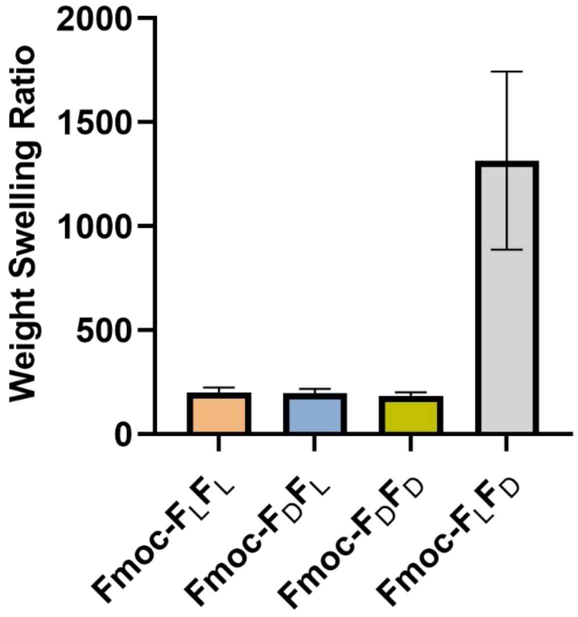

Figure S5. Swelling ratio of four peptide hydrogels.
